# Beliefs, preferences, and informational needs of patients with rheumatoid arthritis and concomitant cancer: a qualitative study

**DOI:** 10.1186/s41927-025-00526-7

**Published:** 2025-07-01

**Authors:** Juan I. Ruiz, Sheneze T. Madramootoo, Maria A. Lopez-Olivo, Namrata Singh, Maria E. Suarez-Almazor

**Affiliations:** 1 Department of Health Services Research, The University of Texas MD Anderson Cancer Center, 1515 Holcombe Boulevard, Unit 1444, Houston, TX 77030 USA; 2 Department of Medicine, Division of Rheumatology, University of Washington School of Medicine, Seattle, Washington, USA

**Keywords:** Rheumatoid arthritis, Cancer, Qualitative research, Decision-making, Diseases-modifying antirheumatic drugs

## Abstract

**Background:**

Treatment of rheumatoid arthritis (RA) with biologic drugs in patients with cancer could potentially result in poor cancer outcomes. This study aimed to identify the beliefs, preferences, and informational needs of patients with RA and cancer regarding the harms, benefits, and uncertainties surrounding the use of RA therapy with respect to cancer.

**Methods:**

We interviewed 20 patients with RA and cancer recruited from a cancer center using a semi-structured guide. We explored patients’ discussions with physicians, beliefs, preferences about RA treatment, and decision-making issues. Using a deductive approach, patients’ responses were grouped according to the explored themes.

**Results:**

Fifteen (75%) patients were women; mean age was 59.9 years (standard deviation, 9.8). Patients discussed RA symptoms, adverse events, drug interactions, and discontinuation of RA treatment after cancer diagnosis; most felt their concerns were clarified after the discussion with their physicians. Some patients were concerned about the risk of cancer development or recurrence due to RA treatment; few were concerned about the interaction between RA and cancer treatment. Patients were concerned about the impact of cancer treatment on RA and potential immunosuppression. Patients relied on discussions with their physician and their own previous experiences to make decisions. Most patients would consider taking a drug for RA even when its impact on cancer is unknown. Patients wanted to receive information about drugs’ efficacy and adverse effects, drug interactions, impact of RA drugs on cancer, and costs.

**Conclusions:**

Our findings on informational needs, concerns, information delivery preferences, and desired level of involvement in the treatment-related decisions of patients with RA and cancer can facilitate the development of educational material that can help with shared decision-making in patients with RA and cancer. We identified important aspects related to the informational needs and concerns of patients with RA and cancer, including worries about not being able to receive RA treatment.

**Clinical trial number:**

Not applicable.

**Supplementary Information:**

The online version contains supplementary material available at 10.1186/s41927-025-00526-7.

## Background

Despite the well-established benefits of disease-modifying antirheumatic drugs (DMARDs) in patients with rheumatoid arthritis (RA), they also have adverse effects related to their immunosuppressant effects, including increased risks of serious infections [1, 2]. There is limited evidence that some DMARDs such as targeted synthetic DMARDs, which include Janus Kinase inhibitors (JAKi), and selected biologic agents such as abatacept may increase the risk of developing malignant tumors [3–5]. Given the potential deleterious impact of these drugs on anti-tumor immunity there have been concerns about their use in patients with RA and concomitant cancer. The evidence regarding the use of DMARDs, more specifically biologic or targeted synthetic DMARDs, in patients with cancer is scarce because patients with cancer are underrepresented in RA clinical trials. The currently available data does not support a deleterious impact of these agents in patients with cancer, but most of the studies examined tumor necrosis factor inhibitors (TNFi), most commonly in patients with breast cancer, and most of the studies included long-term survivors rather than patients with recently diagnosed cancer [6, 7]. Clinical practice recommendations on this topic have been based on expert opinions and low-quality evidence, and do not provide specific guidance that considers type or stage of cancer. For the American College of Rheumatology (ACR) guideline the panel extensively discussed questions concerning the utilization of DMARDs in patients with solid malignancies [8]. However, due to the evolving landscape of personalized treatments for various solid malignancies, the voting panel concluded that formulating a universal recommendation was not feasible. Moreover, the panel stated that while earlier recommendations advised against the administration of TNFi in patients with skin cancer, more evidence is needed to definitively recommend for or against the use of these DMARDs. The European Alliance of Associations for Rheumatology (EULAR) guidelines recommend for patients with poor prognostic factors such as cancer, and who do not achieve RA treatment targets with conventional DMARD, to consider a biologic DMARD or a JAKi, but weighing pertinent risk factors [9]. 

Patients with RA and concomitant cancer may have concerns about their RA drug therapy given the lack of robust guidance, and the uncertainty about the effect of these drugs on cancer outcomes. Although previous studies have evaluated the fears and beliefs about cancer of patients with RA at large, none of them included patients with both RA and cancer [10]. Therefore, there is no direct evidence about this specific subgroup of patients. We conducted a qualitative study to identify the beliefs, preferences, and informational needs of patients with RA and cancer regarding the harms, benefits, and uncertain risks of RA therapy.

## Methods

We followed the Standards for Reporting Qualitative Research reporting guidelines (SRQR) [11]. 

### Qualitative approach

Our theoretical framework was guided by phenomenological theory, which aims to determine the meaning that an experience has to a person by providing a comprehensive description of it [12]. Using this framework, we conducted interviews with patients with RA and cancer to address our research question.

### Research team and reflexivity

The research team consisted of two rheumatologists with expertise in the care of patients with cancer (M.E.S.-A., N.S.), a clinical epidemiologist (J.I.R.), a knowledge synthesis researcher with experience in qualitative research (M.A.L.-O.), and a research coordinator with training in qualitative data collection (S.T.M.). The research team did not have any relationship with the patients who participated in the study, except for one of the rheumatologists who is staff in the clinic where patients were recruited.

### Sampling strategy and context

To recruit participants, we used a purposive sampling approach. Our a priori sample size was 20 participants, which we felt would be sufficient to exhaust all relevant themes and reach thematic saturation, given the narrow scope of the topic and our team’s prior experience in qualitative research [13]. 

Participants were recruited from the University of Texas MD Anderson Cancer Center outpatient clinics and were interviewed between October 2022 and March 2023. We screened patients through the electronic medical record system, and those who met the inclusion criteria were invited to participate by phone. The inclusion criteria were age 18 years or older, diagnosis of RA, diagnosis of any cancer (active or with past history), ability to speak in English, and being in the outpatient setting. All patients had a diagnosis of cancer at MD Anderson and were included in the Tumor Registry. Rheumatoid arthritis was identified based on diagnosis by a rheumatologist at MD Anderson, after review of the electronic medical records. We excluded patients receiving hospice care or admitted for long-term care because these patients were not being seen in the outpatient setting and our interviews concerned outpatient care decisions.

### Data collection instrument and method

We conducted semi-structured interviews with open-ended questions. A guide for the interviews was developed by the research team, based on prior experience in qualitative studies of patients with RA, one of which included patient research partners [14, 15]. Patients were not directly involved in the development of the specific guide for the current study. We explored three major themes: (1) Discussions of patients’ concerns with physicians (rheumatologist and/or oncologist). We asked participants to describe past experiences with medical encounters with respect to discussions about RA treatment after their cancer diagnosis; (2) Beliefs and attitudes about harms and benefits of RA treatment, considering their cancer diagnosis; and (3) Decision-making process and how patients participate in the decisions about RA treatment. A simplified version of the interview guide is shown in the Supplementary Material. The initial guiding questions were the same for all patients but probing varied according to patients’ responses. The interviews were conducted by a female research coordinator with training in qualitative interviews (S.T.M.). At the beginning of the interview, the interviewer described her credentials and the research topic. Throughout the interview, the interviewer encouraged the participants to elaborate on their responses and to provide specific examples when appropriate.

All interviews were conducted by phone, and the duration of interviews ranged from 30 to 45 min. The presence of nonparticipants (e.g., patients’ relatives) was allowed if the participant felt it was helpful. Each interview was audiotaped.

Demographic information and clinical characteristics were collected from the electronic medical records. Results of continuous variables were reported as medians with interquartile ranges and categorical variables were reported as proportions.

### Data processing and analysis

After verbatim transcription of the audiotaped interviews by Landmark Transcription (thelai.com), two analysts (J.I.R., S.T.M.) independently read the transcripts three times to familiarize themselves with the data. We organized, sorted, and interpreted the data using the web application Dedoose, version 9.0.17 (SocioCultural Research Consultants, LLC, Los Angeles, USA) [16]. Data were analyzed using descriptive coding. Descriptive codes were categorized deductively to fit our research questions [17].

### Techniques to enhance trustworthiness

After the first three interviews, two researchers (J.I.R., S.T.M.) met to discuss their findings until consensus regarding the key themes and codes was reached. Subthemes were added and refined with subsequent interviews until a codebook was established. We selected relevant quotations as examples for each subtheme. The codebook was then reviewed iteratively by the remaining investigators (N.S., M.A.L.-O., M.E.S.-A.).

## Results

### Patient characteristics

Out of 58 eligible patients, 20 declined to participate, 18 were not able to be reached after 3 attempts, and the remaining 20 agreed to be interviewed. Patient characteristics are summarized in Table 1. Among the 20 patients, 15 (75%) patients were women, and the mean age was 59.9 years (standard deviation, 9.8). Median time since RA diagnosis was 11 years (range, 1–40). Four (20%) patients had breast cancer, 4 (20%) had melanoma, 2 (10%) had lymphoma, 2 (10%) had other hematologic cancers (myeloproliferative disorder and leukemia), and 1 each (5%) had lung cancer, colon cancer, prostate cancer, uterine cancer, ovarian cancer, oropharynx cancer, thyroid cancer, and neuroendocrine cancer. At the time of the interview, 11 (55%) patients had no evidence of cancer, 3 (15%) had local or regional cancer, and 6 (30%) had metastatic cancer.

**Table 1:** Patient characteristics (*N* = 20)

Characteristic	*n* (%)
**Age**,** median (IQR)**,** years**	59 (55–66)
**Sex**	
Female	15 (75)
Male	5 (25)
**Race**	
Black	4 (20)
White	16 (80)
**Ethnicity**	
Not Hispanic or Latino	14 (70)
Hispanic or Latino	6 (30)
**Marital status**	
Single	2 (10)
Married	15 (75)
Widowed	2 (10)
Separated	1 (5)
**Education level**	
Some high school	1 (5)
High school diploma or equivalent	3 (15)
Trade or technical school	1 (5)
Associate degree	3 (15)
Some college	3 (15)
Bachelor’s degree	6 (30)
Master’s degree	2 (10)
Advanced degree	1 (5)
**Type of cancer**	
Melanoma	4 (20)
Lung	1 (5)
Breast	4 (20)
Colon	1 (5)
Hematologic	4 (20)
Prostate	1 (5)
Uterine	1 (5)
Ovarian	1 (5)
Oropharynx	1 (5)
Thyroid	1 (5)
Neuroendocrine	1 (5)
**Time cancer diagnosis**,** median (IQR)**,** years** **Cancer stage at interview**	3.5 (2–10)
No evidence of disease	11 (55)
Local/regional	3 (15)
Metastatic	6 (30)
**Time from RA diagnosis**,** median (IQR)**,** years**	11 (8–20)
RA treatment received before cancer diagnosis*	
Conventional synthetic DMARDs	15 (75)
Biologic DMARDs	9 (45)
Targeted synthetic DMARDs	2 (10)
Glucocorticoids	3 (15)
None	4 (20)
RA treatment received after cancer diagnosis*	
Conventional synthetic DMARDs	9 (45)
Biologic DMARDs	7 (35)
Targeted synthetic DMARDs	0 (0)
Glucocorticoids	7 (35)
None	3 (15)
Current RA treatment* ^**i**^	
Conventional synthetic DMARDs	8 (40)
Biologic DMARDs	5 (25)
Targeted synthetic DMARDs	2 (10)
Glucocorticoids	5 (25)
None	4 (20)
**Self-reported RA disease activity at cancer diagnosis**	
Active	18 (90)
Inactive	2 (10)

*Some patients received multiple types of RA therapy. ^i^Current refers to the moment of the interview

DMARDs = disease-modifying antirheumatic drugs; RA = rheumatoid arthritis; IQR: interquartile range

Before being diagnosed with cancer, 15 (75%) patients had received conventional synthetic DMARDs, 9 (45%) had received biologic DMARDs, 2 (10%) had received targeted synthetic DMARDs, 3 (15%) had received glucocorticoids, and 4 (20%) had not received any RA treatment. After being diagnosed with cancer, 9 (45%) patients received conventional synthetic DMARDs, 7 (35%) received biologic DMARDs, none received targeted synthetic DMARDs, 7 (35%) received glucocorticoids, and 3 (15%) did not receive any RA treatment. At the time of cancer diagnosis, RA was active in 18 (90%) of the 20 patients, as self-reported by the patients at the time of the interview.

### Response themes

**Fig. 1:**
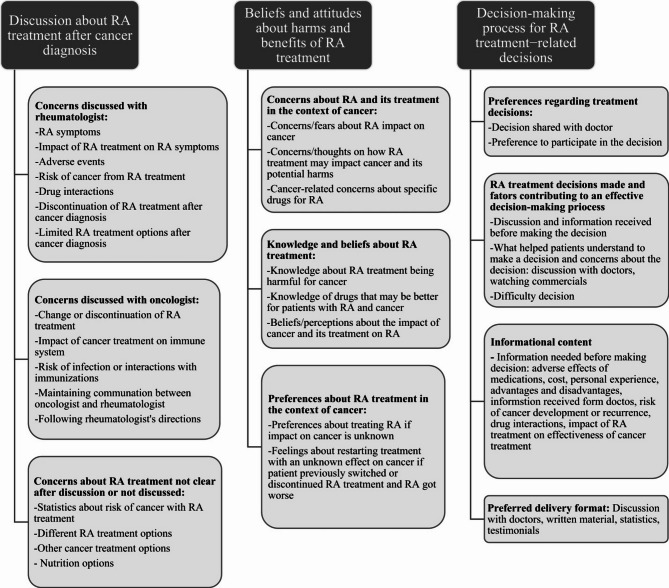
Themes and subthemes from interviews with patients about RA-related treatment decisions

The themes explored, and their respective subthemes are shown in Fig. 1. Thematic saturation was reached with the 20 interviews. Major themes included: (1) Patient-provider communication about RA treatment after cancer diagnosis, (2) Beliefs and attitudes about harms and benefits of RA treatment, (3) Decision-making process for RA treatment decisions.

### Concerns discussed with physicians after cancer diagnosis

With their rheumatologists, patients discussed their concerns about RA symptoms, the impact of RA treatment on RA symptoms, adverse events, including the risk of cancer with RA treatment, and drug interactions (i.e., between RA treatment and immunotherapy and other cancer drugs). A few patients stated that they did not discuss the effect of RA treatment on cancer or the risk of cancer recurrence. Most patients expressed concerns regarding the discontinuation of RA treatment and the limited RA treatment options after their cancer diagnosis.

*Patient 2: “We’ve had discussions about the medication I’m taking and whether the risk of cancer is higher because I’ve had cancer”.*

*Patient 4: “We’ve talked about what doses need to come off during chemo*,* what needs to go back on*,* when chemo’s done and then later*,* as the immune system gets better”.*

*Patient 9: “…they made me aware of that with the cancer I couldn’t take the RA drugs. But as far as other treatments*,* my treatments are limit because of the cancer.”*

Patients discussed similar concerns with their oncologists, including changes or discontinuation of RA treatment and the impact of cancer treatment on the immune system. Participants also discussed the risk of infections, interactions between cancer and RA therapy and immunizations, and the importance of maintaining communication between the oncologist and the rheumatologist regarding treatment interactions. However, a few patients stated that they did not discuss RA treatment in relation to cancer with their oncologist, or that their oncologist suggested that they follow their rheumatologist’s directions.

*Patient 6: “He’s never crossed over between cancer and rheumatoid—and*,* you know*,* and my RA.”*

*Patient 20: “Yeah*,* well*,* during my cancer*,* I was not able to be treated for my RA. I—it went untreated for five years. I wasn’t able to take any treatment for that until I was in the clear as to my cancer.”*

Most patients reported clear understanding after discussions with their providers.

*Patient 7: “Everything was pretty much clear because they described it down to the tee. Which*,* that was understandable. Then*,* they [rheumatologist] drew pictures*,* and that was something different for me.”*

However, some topics were not clear for a few patients, including the potential probability of increased risk of cancer with RA treatment, alternative RA treatment options, nutrition approaches, and other potential cancer treatment options.

*Patient 2: “He wasn’t able to provide any statistics as far as*,* if there’s an increased risk or a frequency of lymphoma due to taking [Etanercept] because I’ve already had cancer. There were no statistics available on that”.*

*Patient 8: “I know that I had quite a few options and medications*,* but we haven’t talked or discussed each one of them carefully”.*

*Patient 10: “I have no fears. RA is—you know—in my opinion*,* it is just one of those background noises because cancer is kind of a dominant issue. So*,* I don’t even consider it an issue at all”*.

### Beliefs and attitudes about harms and benefits of RA treatment

Half of the patients [10] did not express concerns about the potential impact of RA treatment on cancer.

*Patient 1: “I don’t think about it affecting my cancer. I think about it affecting my body. I mean*,* I know that the medications that they use to treat the RA could cause cancer*,* but it’s highly doubtful that it caused the type of cancer that I have”.*

*Patient 6: “I think about them as two separate things…rheumatoid arthritis is one thing*,* and cancer is the other thing. Is something else”.*

*Patient 12: “I don’t have any fears or concerns about that. My doctors—the oncologist and my rheumatologist kept in close contact with one another*,* and so I felt very safe and secure ‘*.

The other half of the patients shared concerns about cancer development, progression, or recurrence, the interaction of RA and cancer drugs, and the lack of efficacy of cancer drugs. A few patients mentioned an increased risk for lymphoma with RA drugs. Two patients stated fears about the impact of cancer on RA symptoms, and one patient expressed concerns about the limited options of cancer treatment because of RA. One patient mentioned concerns about an increased risk of infections due to receiving drugs for RA and cancer simultaneously.

*Patient 9: “That the RA could cause the cancer to get out of control. Like*,* seeing that they both are immune diseases*,* you know*,* I’m sure both of them mess with my immune system.”*

*Patient 13: “I don’t really know what the effects of my RA meds are on my cancer”.*

*Patient 20: “Well*,* there are some medications that are cancer causing*,* so it does bring some concern. But for now*,* I’m not concerned with it*,* so it’s all clear. I’m not concerned any longer at this point”.*

*Patient 16: “I’m just afraid that whatever medication they give me it has a probability of getting cancer—it can come back and—that’s my fear”.*

*Patient 17. “If I had the choice*,* I would rather be treated for cancer versus the RA.”*

Several patients stated they had stopped RA therapy when diagnosed with cancer or starting cancer treatment. When asked about concerns and beliefs related to specific RA drugs in relation to cancer, two patients were concerned about etanercept because of its possible link with cancer; one patient had heard that adalimumab could have negative consequences on cancer outcomes; some patients were concerned about glucocorticoids, not only because of their common adverse effects but also because they cause immunosuppression; and methotrexate because of its adverse effects. However, more than half of the patients did not have concerns about specific RA drugs and a few patients stated that some drugs can negatively impact other cancers but not on their cancer.

When we asked if they knew about specific RA drugs that might be better for patients with cancer, most of them did not know, and a few patients said they wished to know what cancer drugs are better for patients with RA and cancer. Two patients mentioned rituximab as a cancer drug that would be better for patients with RA and cancer, and others stated that the best cancer drug would depend on the type of cancer.

Most patients stated that cancer and/or its treatment negatively affected their RA because they had to discontinue RA treatment and their RA symptoms got worse, or because the cancer treatment made them weak. Five patients said cancer or cancer treatment did not have any impact on RA, and only a few patients believed cancer treatment improved their RA.

Most patients said that they would consider treating their RA even if the impact of RA on their cancer was unknown because they trusted the physician. Quality of life was patients’ main consideration when deciding whether to continue treating their RA, and patients noted that there are always adverse effects related to RA treatment. A few patients said they would need to think more about it.

*Patient 5: “If it’s prescribed and approved by Dr. XXX (oncologist) and the staff*,* I’d take it.”*

*Patient 4: “I would be fine with that. I’m kind—I’m more concerned about the arthritis than I am the cancer. I care about quality of life.”*

Patients had mixed feelings about starting a new RA treatment with an unknown effect on their cancer if their RA got worse, but almost half of the patients said they would consider starting RA treatment in this scenario. Some patients said they would need more information about the effects of RA treatment on cancer before deciding to begin or restart RA treatment. A few patients said whether they would begin treatment would depend on the severity of the RA.

### Decision-making process for RA treatment-related decisions

#### Preferences regarding treatment decisions

Most patients indicated that the recent decision regarding RA treatment was shared with their physician. Most said they would prefer to more greatly discuss the decision with their physician and participate more in the decision-making process, and one said it would depend if the physician believed the discussion is necessary.

*Patient 14: “By both. It’s always been by both because the doctors there do help with get—better*,* but you can address the treatment. And then*,* of course*,* I might then go ahead and respond with my concern or any questions. And I guess together we make a decision as to what’s best for me.”*

#### RA treatment decisions and factors contributing to an effective decision-making process

Patients reported having made decisions about various issues related to RA treatment including: switching treatments, discontinuing treatment because of cancer diagnosis or adverse effects, restarting treatment, and changing dose and route of administration.

*Patient 4: “ we often discuss whether or not to up a dose or decrease a dose or keep it the same and what are my symptoms and are they manageable or not.”*

*Patient 15: “We discussed the steroid shots to help with the joint swelling and pain— and the muscle soreness*,* but I wanted to talk with my cancer doctor before I gave the rheumatologist an answer about using the steroids.”*

To make these decisions, patients reported using information provided primarily from discussions with their physicians, especially about adverse effects, test results, and the advantages and disadvantages of RA treatment during cancer treatment. They also considered their personal experience regarding RA treatment and adverse effects. Only a few patients reported that they sought and obtained information from websites.

*Patient 2: “Just information from my doctor—from my rheumatologist on the side effects—the possible side effects of the medication I was taking.”*

Most patients said that a discussion or an email from their rheumatologist with information helped them understand their options and come to a decision. Reading the drugs information and watching a commercial with information about the adverse effects of a specific RA treatment also helped patients understand the risks of treatment.

This recent decision about RA treatment was not difficult for most patients (*n* = 13, 65%), but a few patients said it was difficult (*n* = 3, 15%). Some patients found the decision difficult and said that the decision was ultimately made by their physician and they trust them (*n* = 4, 20%).

*Patient 7: “Because they explained it to me thoroughly. They broke it down in language terms*,* because some doctors will just use doctors’ terms*,* but they actually broke it down. I mean*,* I call it a dummy term. Medical science for dummies.”*

#### Preferences for delivery of information

Patients believed that receiving information about RA treatment adverse effects before deciding about beginning treatment would be useful, including information about the risk of cancer development or recurrence with RA treatment. A few patients stated that the information received from their rheumatologist and oncologist would be enough. Receiving information about the potential interaction between cancer and RA treatment, and the impact of RA treatment on cancer treatment effectiveness was considered important. Patients also wanted to know when to restart RA treatment if discontinued because of the cancer diagnosis or therapy, as they had concerns about their RA getting worse. Having information on alternative options for treatment was mentioned by a few participants, especially with respect to potential side effects. One patient mentioned wanting information on the cost of treatment. While many of the participants were satisfied with general information from their providers, others wanted more specific data about risks. Only a few patients felt that probability and numerical information were important for their decision-making.

*Patient 2: “I always like to know the side effects and any statistics that they’ve done on the — when they do the trials.”*

*Patient 20: “Making sure that it doesn’t make my cancer worse or my RA worse.”*

In general, patients preferred to receive information about the harms and benefits of RA treatment during a discussion with their physician.

*Patient 4: “I’m just happy with just having a discussion with my doctor”.*

More than half said that it would be valuable to obtain information as written material (e.g., emails, mailings, pamphlets, or through the electronic medical record patient portal).

*Patient 7: “I like pamphlets. I like websites. I like videos. Definitely pictures”.*

Thirteen patients (65%) expressed interest in hearing testimonials from other patients about treatment benefits and risks.

*Patient 12: “Most definitely*,* especially from a person who’s seeing’ it firsthand and what-what has happened to them”.*

One patient felt testimonials would be less relevant due to their personal nature and might not apply to their specific situation.

*Patient 11: “RA is so personal. It’s so*,* yeah*,* it’s just so personal and complicated*,* and it changes all the time from year to year*,* month to month. So I think the testimonials are a very positive spin to whatever therapy you’re doing*,* but I can’t say they’re what make me choose my drug.”*

A few patients (*n* = 3, 15%) preferred actual data to show treatment benefits and risks.

*Patient 1: “I personally like to read the data—the research data that’s been done”*.

## Discussion

To our knowledge, this is the first qualitative study of patients with both RA and cancer, exploring their concerns, beliefs, preferences, and informational needs for treatment-related decision-making. Patients reported discussing their concerns with their physicians. With their rheumatologist, patients discussed concerns, related to RA symptoms, adverse effects of cancer therapy on RA treatment, drug interactions (between RA drugs and cancer treatment such as immunotherapy), and discontinuation of RA treatment. Patients discussed similar concerns with their oncologist, including concerns about the impact of cancer treatment on the immune system. They stressed the importance of communication between specialists. Most patients said that their concerns were clarified after a discussion with their physicians; however, a few patients said some topics were not clear (e.g. statistics on the risk of cancer with RA treatment, and alternative cancer and RA treatment options).

Patients shared their concerns about the risk of cancer development or recurrence and suppression of the immune system. Several were worried about the impact of cancer treatment on RA and RA flares. A systematic review that assessed the fears and beliefs of people with RA reported that most studies described fears related to pharmacological therapy, specifically treatment failure, adverse effects, overprescription of RA drugs, drug interactions, and addiction; none of the studies mentioned a fear of cancer development [10]. However, these studies did not include patients with both RA and cancer.

In another qualitative study conducted to explore beliefs and apprehensions about the disease course and treatment of RA in 25 patients with RA and 25 patients with spondyloarthritis, they expressed fears of increased risk of cancer (especially skin cancer) as an adverse effect of treatments [18]. In our study, half of the patients did not have any concerns about specific RA drugs and their impact on cancer. A few patients mentioned concerns about RA drug effects on cancer or had heard about the link between two tumor necrosis factor inhibitors, etanercept and adalimumab, and cancer, and a few patients mentioned concerns about steroids related to immunosuppression. This perception of increased risk of cancer with tumor necrosis factor inhibitors was also observed in a qualitative study from the United Kingdom that explored the patients’ experiences starting tumor necrosis factor inhibitors [19]. In our study, although patients considered the uncertainty of the risk of cancer development or recurrence, most (70%) said they would consider taking a drug to improve their RA even when the impact of taking such a drug on cancer is unknown.

Regarding the decision-making process about RA treatment, most patients said the decision was shared with their physician and that the decision was not difficult. Patients who were not involved in the decision said they would have liked to participate in it. This finding agrees with the results of a population-based survey that had 2,765 respondents and showed that 96% of participants preferred to be offered choices and asked about their opinions regarding clinical decisions [20]. 

Patients were primarily interested in receiving information about the harms of treatments, potential effects of RA drugs on cancer outcomes and interactions among different treatments. For most patients, the information obtained in the discussion with their rheumatologist and oncologist was sufficient for their decisions. However, there was substantial variation in preferences related to how the information should be delivered. While some patients were satisfied with a general recommendation from their physicians, other were more interested in obtaining numerical and probabilistic data with respect to potential risks. Preferences also varied with respect to delivery of information beyond that offered by their health providers. While many patients felt peer testimonials would be helpful, other felt these would not be relevant to them or could even be detrimental. Other identified delivery tools, included mailings, emails, websites, electronic patient portal messages or videos, as useful sources of information, but these varied across patients [21, 22]. 

As patients preferred mode of obtaining information was direct communication with providers, guidance is needed so clinicians in the community can provide reliable information to patients. Consensus recommendations jointly developed by rheumatologists and oncologists, based on evidence when available - and expert opinion recognizing uncertainties when evidence is lacking - can support shared decision-making in patients with RA and cancer. A recent effort by the European Alliance of Associations for Rheumatology (EULAR) is a step in this direction [21, 22]. 

Our study has some limitations. While the interview guidance and initial themes were based on prior qualitative studies of patients with RA, we did not fully involve participants throughout the research process. A more prolonged engagement of participants and meaningful involvement from design to analysis would have increased the trustworthiness of the findings. As our interviews were guided by specific themes, we did not conduct an in-depth exploration of other contextual information that could assist in the applicability of findings to other settings. However, our sample was broad with respect to participant characteristics and clinical status. This information is provided, and we have also included *verbatim* quotes which can aid readers in determining the transferability of our findings to other settings. Finally, we conducted all interviews by phone to enhance patient convenience and safety, as we were at the end-tail of the COVID-19 pandemia; in-person communication could have been richer and also provide non-verbal information.

Our findings emphasize the importance of communicating risks and uncertainties tailored to individual preferences for delivery of information. While almost every patient expressed a preference for direct information from their providers, many also believed that other tools would be helpful. These aids varied from peer testimonials to visual aids for probabilistic presentation of harms; therefore, communication may be more effective if personalized. Multimedia tools offering access to narrative information, quantitative data in graphs, or patient testimonials, could aid patients obtaining information through a preferred delivery mode, and support informed discussions with their providers.

## Conclusions

Our study shows variation in patients’ knowledge, beliefs, and attitudes about the treatment of RA since they develop cancer. Patients expressed concerns about whether their RA treatment might increase their risk of having their cancer progress or return. They worried about how RA and cancer treatments might interact. They were also concerned about how cancer treatment might affect their RA. Ultimately, patients made decisions about their care based on conversations with their doctors and their own past experiences. These findings can enhance discussions between healthcare providers and their patients, as they provide a deeper understanding of the preferences and informational needs of patients with RA and cancer and can assist in the development of educational materials to help with shared decision-making.

## Electronic supplementary material

Below is the link to the electronic supplementary material.

Supplementary Material 1

Supplementary Material 2

## Data Availability

Data generated or analyzed during this study are included in this published article and its supplementary information or are available from the corresponding author on reasonable request.

## Abbreviations

RA: Rheumatoid arthritis
DMARD: Disease-modifying antirheumatic drugs
JAKi: Janus kinase inhibitors
TNFi: Tumor necrosis factor inhibitors
ACR: American college of rheumatology
EULAR: European alliance of associations for rheumatology
SRQR: Standards for reporting qualitative research
