# Supplementary material for: Beliefs, preferences, and informational needs of patients with rheumatoid arthritis and concomitant cancer: a qualitative study
Source: BMC Rheumatol. 2025 Jul 1;9:79. doi: 10.1186/s41927-025-00526-7 (PMC12220157; doi:10.1186/s41927-025-00526-7)
Supplement: Supplementary file 1 — Supplementary Material 1 [file 41927_2025_526_MOESM1_ESM.docx]

**Supplementary Material**

1. **Guided interview for patients**

**THEME 1. Past experience with medical encounters.**

1. Could you describe what discussions you have had, if any, with your rheumatologist with respect to your RA treatment?

2. Could you describe what discussions you have had, if any, with your oncologist with respect to your RA treatment?

3. What concerns about the treatment of your RA did you share with your doctors?

4. What issues or concerns about RA treatment were still not clear to you after the discussions with your doctors?

5. Are there any other concerns about RA treatment that you did not get a chance to discuss with your doctors?

**THEME 2. Beliefs and attitudes about harms and benefits of RA treatment.**

1. What concerns or fears do you have about how your RA might affect your cancer?

2. What are your thoughts on how the treatment of RA may impact your cancer? What potential harms are you concerned about?

3. Are you concerned about taking any specific drugs used for the treatment of RA, now that you have cancer?

4. In what ways has cancer and its treatment affected your RA?

5. Think about your cancer and your RA, and your overall wellbeing. For some RA treatments we may not know if they can make the cancer worse or not.

a. How would you feel about treating your RA with these drugs that may improve your arthritis but that we don’t know if they can make your cancer worse?

b. If you changed or stopped your RA treatment, and your arthritis got worse, how would you feel about starting treatment if we don’t know what effect these drugs may have on your cancer?

**THEME 3. Decision-making process.**

1. Are the decisions related to RA treatment made primarily by your doctor telling you what to take, by you after listening to your doctor, or shared, by you and your doctor together?

2. Can you give an example of how you made a recent decision about RA treatment?

a. What information did you have before making the decision?

b. What helped you understand the information?

c. What concerns did you have?

d. How difficult was it for you making the decision? What helped?

3. What other types of information would you like to have before making decisions about receiving RA treatment?
